# Editorial: Insect physiology aspects of environmentally friendly strategies for crop pests and invertebrate vector control, volume II

**DOI:** 10.3389/fphys.2025.1601424

**Published:** 2025-04-15

**Authors:** Marcelo Salabert Gonzalez, Guenter Artur Schaub, Norman Arthur Ratcliffe, Ana Claudia do Amaral Melo

**Affiliations:** ^1^ National Institute of Science and Technology - Molecular Entomology, Rio de Janeiro, Brazil; ^2^ Department of General Biology, Institute of Biology, Fluminense Federal University, Niterói, Brazil; ^3^ Postgraduate Program in Science and Biotechnology, Fluminense Federal University, Niterói, Brazil; ^4^ Postgraduate Program in Applied Physics, Institute of Physics, Federal University of Rio de Janeiro, Rio de Janeiro, Brazil; ^5^ Chair of Evolutionary Ecology and Animal Biodiversity, Working Group for Zoology and Parasitology, Ruhr-Universität Bochum, Bochum, Germany; ^6^ Department of Biosciences, Swansea University, Swansea, United Kingdom; ^7^ Chemistry Institute, Federal University of Rio de Janeiro, Rio de Janeiro, Brazil

**Keywords:** insect physiology, crop pests, insect vectors, environmentally friendly strategies, insect control

The articles featured in this second volume explore innovative and sustainable strategies for controlling insect pests and disease vectors, drawing on a deep understanding of insect physiology. The studies presented here encompass a wide range of techniques, from the use of natural control agents (biocontrol) to advanced molecular tools aimed at managing insect populations. Each strategy explores aspects of the survival, morphology, behavior, and physiology of the insect models studied, intending to develop environmentally safe solutions that reduce reliance on chemical agents both in agriculture and in the control of disease vectors, thereby minimizing environmental impact.

The study by Vivekanandhan et al. demonstrates the effectiveness of the natural oil, Cajeput, as a control agent against *Anopheles stephensi*, one of the malaria vectors. The study revealed that Cajeput oil has larvicidal properties, acting by directly inhibiting the carboxylesterase and acetylcholinesterase enzyme systems of *A. stephensi* larvae. Furthermore, the authors demonstrated that there are no significant toxicity risks to non-target species, such as the earthworm *Eudrilus eugeniae*. Based on the results obtained, the authors suggest replacing the widely used chemical agents in malaria mosquito control with essential oils as a correct, natural, and eco-friendly alternative for controlling malaria incidence.

Costa et al. explore the ability of triatomine bugs, vectors of Chagas disease, to feed on sugar as a potential strategy for controlling transmission. Through artificial feeding experiments, researchers reported the ingestion of sugar solutions in several species of triatomines. They also showed that when first instar nymphs of *Rhodnius prolixus* ingested different combinations of insecticides with sugar, survival was significantly reduced. This discovery supports the idea of using sugar-based traps combined with chemical agents to reduce triatomine populations and lower Chagas disease transmission.

This second volume also introduces research by Rouyar et al. on the neural mechanisms of olfaction in *Aedes aegypti*, a major vector of arboviruses worldwide. By developing a transgenic strain of *A. aegypti*, the authors investigated the role of GABA-type receptors in odor detection, shedding light on the genetic and neural processes underlying attraction to odors. The observed changes in attractiveness to fruit scents emphasize the importance of the GABA-B1 receptor in mosquito olfaction. These findings could help us create more targeted and effective strategies for mosquito control, making a real difference in managing these vectors.

Another study by Duan et al. featured here examines *Bactrocera dorsalis*, the oriental fruit fly, and the attraction effect of L-prolinamide, a metabolite isolated from *Enterobacter cloacae*, a gut bacterial strain from *B. dorsalis*. In contrast to most of the current attractants, there was no significant difference between the attraction effect of L-prolinamide on male and female adults. The attraction mechanism of *B. dorsalis* to *E. cloacae* and its metabolites provides new prospects for the development of novel green control technologies for this notorious pest.

Guo et al. present details of how *Riptortus pedestris* (Fabricius) (Hemiptera: Alydidae), a major soybean pest throughout East Asia, relies on its advanced odorant-binding proteins (OBPs) to detect plant-derived volatile compounds produced by soybeans. Understanding OBP interactions with specific volatile compounds could lead to new ecologically friendly pest management approaches, disrupting feeding and resulting in more targeted and efficient control methods for crop pests.

Another interesting study by Yi et al. also investigates OBPs, but in *Picromerus lewisi*, a natural predator of agricultural pests. Through transcriptomic analysis, the authors identified 15 OBPs from this species. They also observed sex-dependent differences in gene expression after exposure to odors from *Spodoptera litura*-infested tobacco plants. These results suggest that some OBPs play a pivotal role in detecting herbivore-induced plant volatiles. Based on this, it is possible to develop pest management strategies that attract beneficial predators.

Research by Sankar et al. reports on the combination of diflubenzuron, an insect growth regulator that interferes with chitin synthesis, with verapamil, a calcium channel blocker, in order to control *Aedes aegypti*. The results show that this combination enhances the effectiveness of diflubenzuron by reducing adult emergence while having no harmful effects on non-target organisms. This indicates a safer and more sustainable approach to mosquito population control by developing targeted and eco-friendly strategies for managing *Ae. aegypti* populations more effectively.

Lanzaro et al. present their results of a study on Cry1Ac toxin binding in the velvetbean caterpillar, *Anticarsia gemmatalis*, a soybean pest in Brazil. The study explored the role of midgut aminopeptidases N (APNs) as receptors for Cry1Ac, using immunohistochemistry, ligand blotting, and mass spectrometry to identify seven APNs involved in toxin interaction. The authors propose that these findings contribute to our understanding of Cry toxin mechanisms and resistance management in *A. gemmatalis*, supporting the development of more effective biopesticides.

A comprehensive review is also presented by Izadi of diapause regulation in insects, a key survival mechanism allowing them to endure stressful environmental conditions Approximately 250 papers were analyzed to consolidate current knowledge on the enzymatic and hormonal regulation of diapause. The review also lays the groundwork for enhancing pest control strategies and ecological conservation by deepening our understanding of diapause mechanisms. By analyzing the hormonal and enzymatic pathways involved in diapause, the study provides valuable knowledge that could pave the way for novel pest control strategies.

The studies presented here reveal the growing body of knowledge related to environment friendly approaches that can be used for pest and vector management. By integrating innovative biocontrol techniques, molecular tools, and the physiological aspects of insect survival, this volume represents a significant step toward more effective and ecologically sound insect control strategies.

Research in the field of entomology and insect physiology must continue to be funded so that we can expand our knowledge and develop more sustainable methodologies for controlling pest insects and disease vectors, aiming to achieve ecological balance and the health of all living beings on Earth.

